# Relationship model between surface strain of concrete and expansion force of reinforcement rust

**DOI:** 10.1038/s41598-021-83376-w

**Published:** 2021-02-18

**Authors:** Fanxiu Chen, Zuquan Jin, Endong Wang, Lanqin Wang, Yudan Jiang, Pengfei Guo, Xinya Gao, Xiaoyuan He

**Affiliations:** 1 School of Science, Qingdao University of Technology, Fushun Road 11#, Qingdao, 266033 Shandong China; 2 School of Civil Engineering, Qingdao University of Technology, Qingdao, 266033 Shandong China; 3 Sustainable Construction, The State University of New York, Syracuse, NY 13210 USA; 4 Department of Engineering Mechanics, Southeast University, Nanjing, 210096 Jiangsu China

**Keywords:** Structural materials, Theory and computation

## Abstract

Concrete cracking caused by corrosion of reinforcement could significantly shorten the durability of reinforced concrete structure. It remains critical to investigate the process and mechanism of the corrosion occurring to concrete reinforcement and establish the theoretical prediction model of concrete expansion force for the whole process of corrosion cracking of reinforcement. Under the premise of uniform corrosion of reinforcing steel bars, the elastic mechanics analysis method is adopted to analyze the entire process starting from the corrosion of steel bars to the cracking of concrete due to corrosion. A relationship model between the expansion force of corrosion of steel bars and the surface strain of concrete is established. On the cuboid reinforced concrete specimens with square cross-sections, accelerated corrosion tests are carried out to calibrate and verify the established model. The model can be able to estimate the real-time expansion force of reinforced concrete at any time of the whole process from the initiation of steel corrosion to the end of concrete cracking by measuring the surface strain of concrete. It could be useful for quantitative real-time monitoring of steel corrosion in concrete structures.

## Introduction

In marine and coastal regions, the existence of chloride ions in humid environments causes the steel bars buried in concrete to be gradually blunt and turning rusty, imposing serious impacts on reinforced concrete structures. Over the past decade, a significant number of reinforced concrete structures failed within their expected service-life ranges due to this so-called corrosion phenomenon. During these corrosion processes, the volumes of the rusted products turn larger than those of the original corresponding reinforcement bars leading to expansion forces to the surrounding. This kind of forces occurring at the interfaces between reinforcement bars and concrete elements due to expansion is termed as steel rust expansion force^1,2^. Rust expansion force causes concrete cover to crack along reinforcement bars, which renders the reinforcement bars directly exposed to external environments, and dramatically reduces the protective capability of concrete to the reinforcing bars. Subsequently, it could further aggravate the corrosion process of steel bars, and induce the peeling of protective concrete layer, and eventually lead to the structural failure of corresponding systems. From this perspective, it is vital to investigate the corrosion and rust expansion process of concrete reinforcement to establish theoretical models for characterizing its underlying mechanism and inherent relationships.

Many scholars studied the physical phenomena of rust expansion cracking in concrete structures and attempted to quantify the rust expansion force of steel bars^3^ using three diverse approaches including theoretical analysis, experimental investigation and numerical simulation. Several theoretical models and functions were introduced to the understanding of the rust expansion cracking process and the relevant mechanisms in reinforced concrete structures. Based on the elasticity theory, Lu et al.^4^ put forward the calculation formula of rust expansion force right before and exactly at the instant moments of the expansion of surrounding concrete covers, assuming uniform corrosion of reinforcement. They discussed several influencing factors related to the expansion force of steel rust. Šavija et al.^5^ used a two-dimensional lattice model to understand the cracking mechanisms resulting from the process of reinforcement corrosion. The heterogeneous properties of concrete were considered for their mechanical investigation and analysis. Zhao et al.^6^ applied Gaussian function to the investigation of non-uniform corrosion process and proposed a multi-point corrosion model. They found that the location of the greatest corrosion is dependent on the pathway how the corrosives reach to the reinforcements and the concrete crack occurs near the point with the greatest corrosion degree. Supplementary to the theoretical analysis approach, multiple laboratory tests were carried out based on acoustic emission and optical measurement techniques. Zaki et al.^7^ adopted acoustic emission technology to understand the corrosion of steel bars. Their experiments showed that the acoustic emission technology can effectively monitor the preliminary process of corrosion associated with the reinforced concrete structure at an initial phase. Kashani et al.^8^ took advantage of a three-dimensional optical measurement technology to characterize the three-dimensional corrosion morphology and patterns of reinforcing elements under accelerated corrosion situations. They constructed a list of probability distribution models for characterizing the geometric features of the corroded reinforcement components. Alhozaimy et al.^9^ implemented a series of laboratory tests to explore the mechanism related to the high corrosion phenomenon occurring at bar intersections. During the study, the related parameters, including half-cell potential, corrosion current, concrete resistivity, were derived from laboratory measurements. Torres-Acosta^10^ conducted experiments to find the critical level of bar corrosion for cracking concrete covers. The level was empirically projected by accounting specimen dimensions. Progresses and magnitudes of stresses due to corrosion were measured and quantitatively determined based on specimen sizes and material properties. With advances in computer technology, finite element analysis has been increasingly adopted for the numerical simulation of reinforcement corrosion process. Based on regular finite-element analysis technique, Molina et al.^11^ proposed a numerical method to simulate the cracking process in concrete subject to corrosion of reinforcement. These colleagues applied the so-called smeared-crack approach for investigating finite elements’ behavior, and integrated initial strains and property change to model the corrosion. Fernandez et al.^12^ combined three-dimensional finite element analysis and three-dimensional optical measurement technique to explore the mechanical impacts of corrosion surface pits on steel bars. Three-dimensional optical measurements were utilized for the acquisition of the related geometry information. Finite element analysis model was established for the understanding of failure course and the measuring of local impacts on the pertinent pits. The local influences of pits on corrosion mechanical properties with single and cyclic loading scenarios were examined. Redaeli et al.^13^ took the finite element analysis technique to characterize the chloride induced corrosion of reinforcing bars in concrete structure members. Ožbolt et al.^14^ proposed a 3D chemo-hygro-thermo-mechanical model to characterize the interaction among the non-mechanical factors and the mechanical features related to concrete. Two numerical examples were presented to illustrate the usage of the model.

The existing theoretical models of expansion force of reinforcement bars generally lack a comparison with experimental data or a result verification process due to the difficulty in obtaining the experimental data on the expansion force of reinforcement bars. By combining theoretical analysis and experimental verification, this paper intends to analyze the whole rusting process of reinforcement bars under the condition of uniform corrosion. The accelerated corrosion test results are compared and cross-validated with those received from the analysis with the theory of elasticity. A relationship model between the rust expansion force of reinforcement and the surface strain of concrete is built, calibrated, and verified.

## Model derivation

### Calculation diagram of the test piece

**Figure 1:**
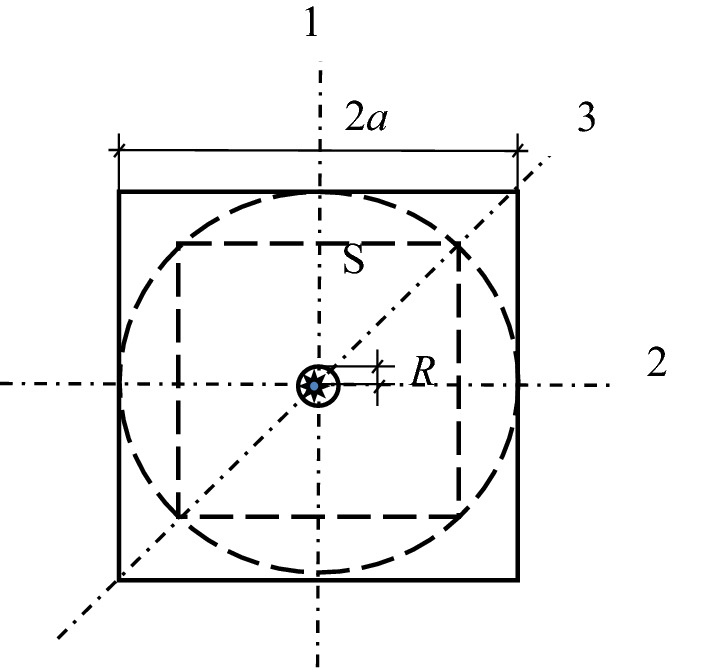
Calculation sketch partition of the reinforced concrete specimen.

**Figure 2:**
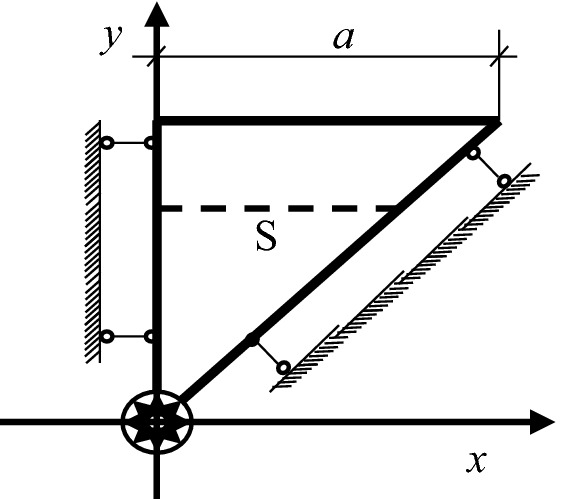
Simplified symmetrical computation sketch of the specimen.

**Figure 3:**
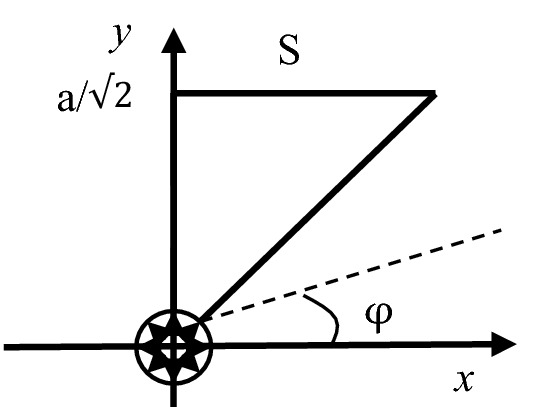
Decomposition of the lower half coordinate system on the complete contact theory.

**Figure 4:**
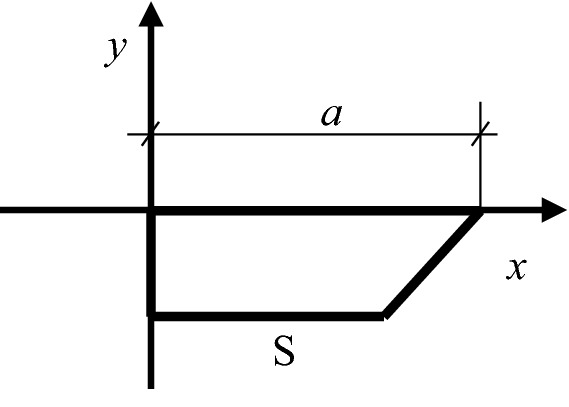
Decomposition of the upper half coordinate system on the complete contact theory.

Taking an squared cross section of the reinforced concrete which has a side length of 2a as the research object, it assumes that there is a reinforcement bar with the radius of R pointing to the center of the cross section and the uniform rust expansion force produced by the steel bar to be q. In order to combine the theory of complete contact of elasticity and Lame's solution, it firstly finds the largest inscribed circle for the square section of the specimen, and then locates the largest inscribed square S of the obtained circle. As in Fig. 1, the square S shown in the dotted line is the largest inscribed square nested by the largest inscribed circle. It can be seen that this section has three axes of symmetry, including axes 1, 2 and 3. Then, Fig. 2 simplifies the calculation sketch considering these symmetries of the test piece. As shown in Fig. 3 and Fig. 4, taking S-plane as the partition, the calculation diagram can be divided into two parts (the upper and the lower) for analysis. Coordinate systems are established, respectively. Besides, the S-plane boundaries in Figs. 3 and 4 fulfill the full contact condition of elastic mechanics. Figure 3 is to calculate the stress and strain on the S-plane in the polar coordinate system by using Lame’s solution, and to convert the stress and strain to the boundary condition on the S-plane in the rectangular coordinate system through the coordinate transformation as shown in Fig. 4. Meanwhile, Fig. 4 is to establish the relationship model between the expansion force of reinforcement rust and the surface strain of concrete specimen by adopting the complete contact theory of elasticity in the rectangular coordinate system.

### Relationship between the expansion force of steel rust and the surface strain of concrete

In the coordinate system of Fig. 4, the semi inverse solution of elasticity is adopted. According to the boundary conditions, ${{(\sigma }_{y})}_{y=0}=0$ is derived. However, with the increase of *y*, σ_y_ deviates from 0. Therefore, assuming *σ*_*y*_ = f_1_(*y*)g_1_(*x*)q, the stress function and stress components are obtained as Formula (1) and Formula (2).

1 $$\begin{gathered} \Phi = [(Fy^{3} { + }Gy^{2} + Hy + I)(Bx^{3} + Cx^{2} + Dx + E) - x(\frac{3}{5}BFy^{5} { + }BGy^{4} + \frac{1}{6}Jy^{3} + \frac{1}{2}Ky^{2} + Ly) - \hfill \\ (\frac{1}{5}CFy^{5} + \frac{1}{3}CGy^{4} + \frac{1}{6}Ny^{3} + \frac{1}{2}Dy^{2} )]q \hfill \\ \end{gathered}$$

2 $$\left\{ \begin{gathered} \sigma_{y} { = }\frac{{\partial^{2} \Phi }}{{\partial x^{2} }} = [(Fy^{3} { + }Gy^{2} + Hy + I)(6Bx + 2Cx)]q \hfill \\ \sigma_{x} { = }\frac{{\partial^{2} \Phi }}{{\partial y^{2} }} = [(6Fy{ + 2}G)(Bx^{3} + Cx^{2} + Dx + E) - x(12BFy^{3} { + 12}BGy^{2} + Jy + K) - \hfill \\ (4CFy^{3} + 4CGy^{2} + Ny + P)]q \hfill \\ \tau_{xy} = - \frac{{\partial^{2} \Phi }}{\partial y\partial x} = - [(3Fy^{2} { + 2}Gy + H)(3Bx^{2} + 2Cx + D) - (5BFy^{4} { + 4}BGy^{3} + \frac{1}{2}Jy^{2} + Ky + L)]q \hfill \\ \end{gathered} \right.$$

In Fig. 4, the upper and lower boundaries are the main boundaries because the transverse part is larger than the longitudinal part. The main boundary conditions are: (σ_y_)_y=0_ = 0, (τ_xy_)_y=0_ = 0, (τ_xy_)_x=0_ = 0. The stress and strain of the S-plane in Fig. 3 are transformed into the boundary conditions of the S-plane in the rectangular coordinate system as in Fig. 4. The final stress function and stress component are shown in Formula (3).

3 $$\left\{ {\begin{array}{*{20}l} {{\upsigma }_{{{\rm y}}} = \frac{{\partial^{{2}} {\Phi }}}{{\partial {\text{x}}^{{2}} }} = \left( {\frac{{{0}{\text{.2R}}^{{2}} {\text{y}}^{{3}} }}{{{\text{a}}\left( {{\text{a}}^{{2}} - {\text{R}}^{{2}} } \right)\left( {{1} - \frac{{1}}{{\sqrt {2} }}} \right)}} + \frac{{{1}{.2\text{R}}^{{2}} {\text{y}}^{{2}} }}{{{\text{(a}}^{{2}} - {\text{R}}^{{2}} {)}}}} \right)\left( { - \frac{{{\text{2x}}}}{{{\text{a}}^{{2}} }}} \right){\text{q}}} \hfill \\ {{\upsigma }_{{{\rm x}}} = \frac{{\partial^{{2}} {\Phi }}}{{\partial {\text{y}}^{{2}} }} = \left[ {\begin{array}{*{20}c} {\left( {\frac{{{1}{\text{.2R}}^{{2}} {\text{y}}}}{{{\text{a(a}}^{{2}} - {\text{R}}^{{2}} {)(1} - \frac{{1}}{{\sqrt {2} }}{)}}} + \frac{{{2}{\text{.4R}}^{{2}} }}{{\left( {{\text{a}}^{{2}} - {\text{R}}^{{2}} } \right)}}} \right)\left( { - \frac{{1}}{{{\text{a}}^{{2}} }}{\text{x}}^{{2}} + {1}} \right) + \frac{{{0}{\text{.8R}}^{{2}} {\text{y}}^{{3}} }}{{{\text{a}}^{{3}} {\text{(a}}^{{2}} - {\text{R}}^{{2}} {)(1} - \frac{{1}}{{\sqrt {2} }}{)}}} + \frac{{{4}{\text{.8R}}^{{2}} {\text{y}}^{{2}} }}{{{\text{a}}^{{2}} {\text{(a}}^{{2}} - {\text{R}}^{{2}} {)}}}} \\ \end{array} } \right]q} \hfill \\ {{\uptau }_{{{{\rm xy}}}} = - \frac{{\partial^{{2}} {\Phi }}}{{\partial {\text{y}}\partial {\text{x}}}}{ = }\left( {\frac{{{0}{\text{.6R}}^{{2}} {\text{y}}^{{2}} }}{{{\text{a}}\left( {{\text{a}}^{{2}} - {\text{R}}^{{2}} } \right)\left( {{1} - \frac{{1}}{{\sqrt {2} }}} \right)}} + \frac{{{2}{.4\text{R}}^{{2}} {\text{y}}}}{{\left( {{\text{a}}^{{2}} - {\text{R}}^{{2}} } \right)}}} \right)\left( {\frac{{{\text{2x}}}}{{{\text{a}}^{{2}} }}y} \right){\text{q}}} \hfill \\ \end{array} } \right.$$

Putting Formula (3) into the physical formula of elastic mechanics^15^, the relationship model between concrete surface strain and steel rust expansion force can be obtained as Formula (4), in which *E*_*c*_ is the elastic modulus of concrete.

4 $$q=\frac{{\varepsilon }_{x}\left({a}^{2}-{R}^{2}\right){a}^{2}{E}_{c}}{2.4{R}^{2}({a}^{2}-{x}^{2})}$$

Concrete materials are neither completely uniform nor fully elastic, and concrete mix proportion and aggregate particle size can affect corrosion process and relevant parameters^16^. The corrosion of steel bars caused by material heterogeneity and environmental factors is non-uniform, so Formula (4) needs to be calibrated to be Formula (5), where *k* is the calibration coefficient.

5 $$q=\frac{{k\varepsilon }_{x}\left({a}^{2}-{R}^{2}\right){a}^{2}{E}_{c}}{2.4{R}^{2}({a}^{2}-{x}^{2})}$$

In the formula, *q* is the rust expansion force of reinforcement, ε_x_ is the strain along the x direction of concrete surface, *a* is half of the side length of the surface of concrete specimen, *R* is the radius of reinforcement, E_c_ is the elastic modulus of concrete, and *k* is coefficient of correction.

## Accelerated corrosion test

In order to verify the established model and determine the value of the correction coefficient *k*, accelerated corrosion test is carried out to obtain the data of the concrete surface strain and the expansion force of reinforcement rust. The surface strain of concrete is measured by the strain gauges attached onto the concrete surface. During the test, steel tube is used to substitute reinforcement bar to be embedded into concrete, and the strain gauges are arranged onto the inner wall of the steel tube along its circumferential direction. The rust expansion force caused by the corrosion of the outer wall of the steel tube is calculated by measuring the circumferential strain of the inner wall and treated as the referential expansion force of rust.

### Relationship between the strain of the inner wall of steel pipe and the expansion force of rust

According to Lame's solution of elasticity^15^, the relationship between the expansion force of the outer wall rust and the circumferential strain of the inner wall of the steel tube is shown as follows.

6 $$q = (1 - \sqrt {1 - \varepsilon_{hs} } )\frac{{E_{s} (R^{2} - r_{1}^{2} )}}{{2\mu R^{2} }}$$

7 $$\varepsilon_{hs} = \frac{{r_{1}^{2} - r_{2}^{2} }}{{r_{1}^{2} }}$$

According to the theory of force and reaction force, the magnitude of the rust expansion force acting on the outer wall of the steel pipe is equal to that of the steel pipe but having an opposite direction. Therefore, in Formula (6) and (7), *q* still represents the rust expansion force of the outer wall of the steel pipe. *E*_*s*_ is the elastic modulus of the steel pipe. μ is the Poisson's ratio of the steel pipe. *r*_1_ is the inner radius before the deformation of the steel pipe. *r*_2_ is the inner radius after the deformation of the steel pipe. *R* is the outer radius of the steel pipe.

### Test preparation

**Figure 5:**
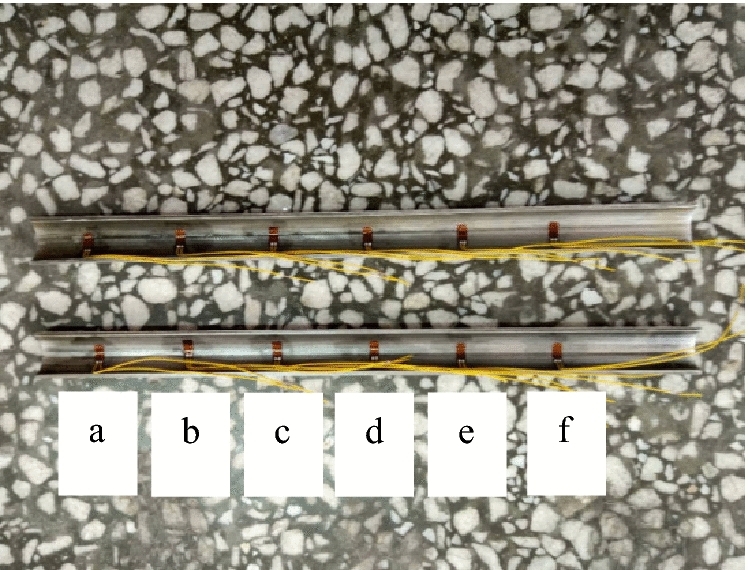
Positions of strain gauges on inner wall of tube.

**Figure 6:**
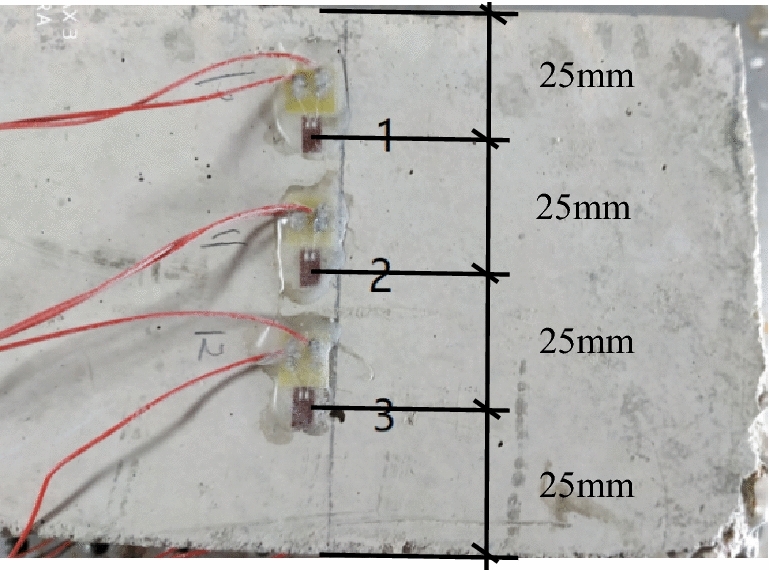
Positions, layouts and sequences of strain gauges.

**Figure 7:**
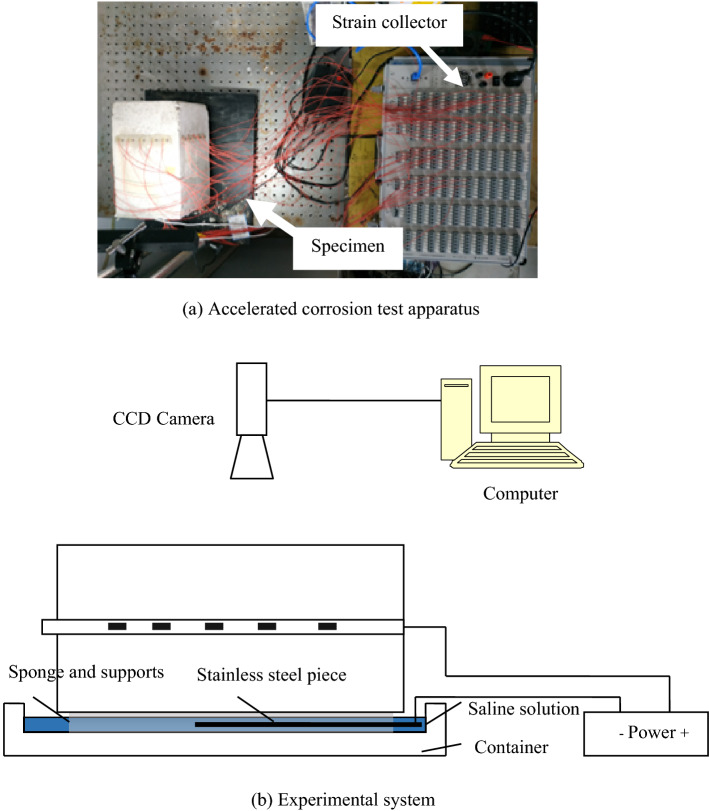
Experimental measurement system.

C30 composite Portland cement and the clean river sand with particle sizes ranging between 5–10 mm are used for the test. Concrete proportioning is shown in Table 1. The coarse aggregate sizes are minimized to avoid their negative impacts on test results. River sand is selected as the fine aggregate. River sand is washed and air-dried to eliminate the influences of chloride in the sand on test accuracy. Tap water is used during the test with sodium chloride added and the concentration is 2‰. Then, the reinforcement is replaced by a hollow steel pipe with its outer diameter of 16 mm and inner diameter of 10 mm. the steel pipe is longitudinally cut into two halves and polished. The inner wall is then adhered with strain gauges along its ring direction, as shown in Fig. 5. While point C is 70 mm away from the end of the steel pipe, point D is located at the pipe middle. Besides, the 703 glue is applied to the strain gauges for protective purpose. A wire is tied to the steel pipe for supplying power to the accelerated corrosion test. Two steel pipe halves are then bonded with epoxy resin. The obtained new steel pipe is put into a 100 mm × 100 mm × 300 mm mold with concrete being poured. Subsequently, the entire mold is placed on a vibration table to be vibrated for 5 min. After standing for 24 h, the concrete piece is remolded and placed into a dedicated room for steam curing. Before the test, three strain gauges are attached to the positions which are 70 mm from the longitudinal edges of the reinforced concrete specimen to obtain the concrete surface strain. Strain gauge positions, spacing layouts and their sequencing numbers are shown in Fig. 6. The strain gauges on the inner wall are wired to a strain collector. The voltage for the accelerated corrosion test is 30 V. The electricity current is periodically recorded. The accelerated corrosion test is set up in Fig. 7. During the test, Basler CCD camera is used to collect the real-time information of the concrete surface strain. The strain collector is used to collect the strain data related to the inner wall of the steel tube and the concrete surface simultaneously.

**Table 1:** Concrete mixture proportion.

Constituent	Cement	River sand	Gravel	Water	Salt concentration
Consumption	480 kg	730 kg	1 095 kg	155 kg	2%

## Analysis of the test results

### The pattern of concrete surface strain versus time

**Figure 8:**
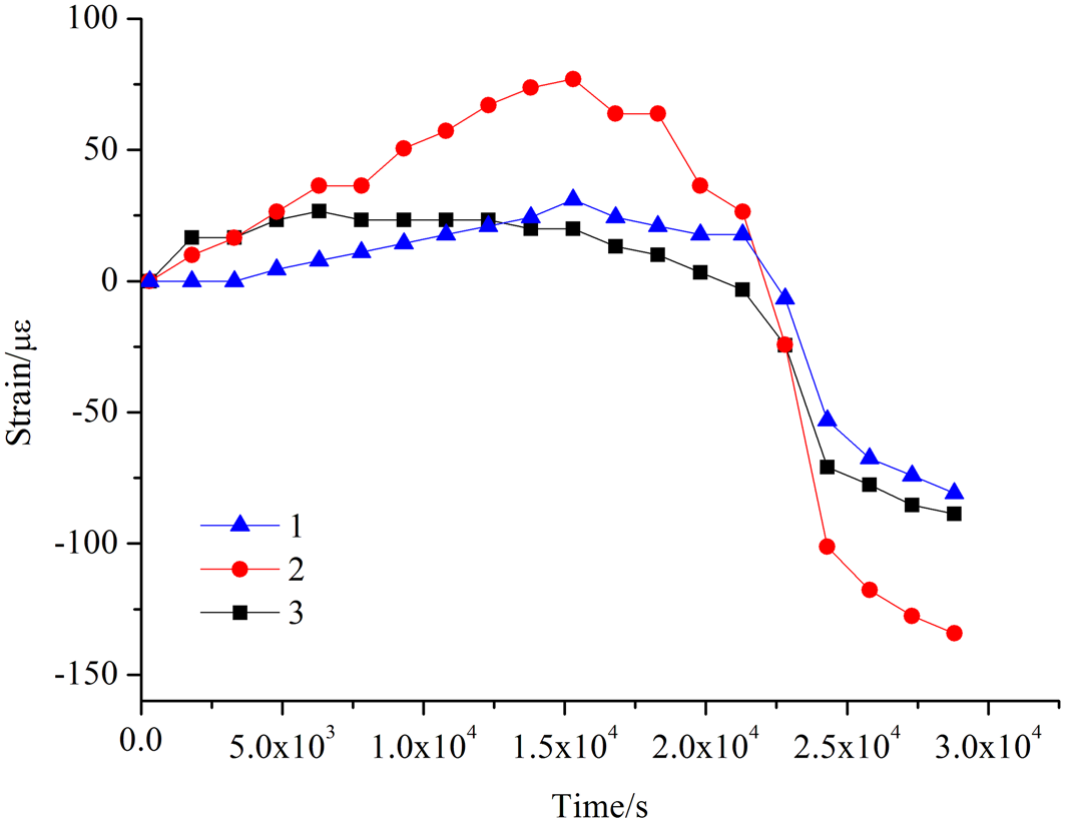
Strain–time relationship for concrete surface.

The variation patterns of strain versus time corresponding to the 1–3 measuring points on the concrete surface are shown in Fig. 8. After the accelerated corrosion test, all the three test points 1, 2 and 3 on the concrete surface are in tensile state, and their tensile strains increase with time. When t = 1.5 × 10^4^ s, cracks appear at the contacting interface of the concrete and the steel tube. After that moment, the bounding force of concrete on the steel tube decreases and the tensile strains at the three measuring points grow at lower rates and gradually turn into declining mode, but these points are still in tension state. Until t = 2.2 × 10^4^ s, the concrete piece cracks completely, and its surface transits from being tensile to be compressive. Measurement point 2 lies at the middle of the concrete surface and its measured strain is larger than those collected at the same time from points 1 and 3. Measurement points 1 and 3 are symmetric, and their strains are roughly equal in terms of magnitude.

### The pattern of rust expansion force of reinforcement versus time

**Figure 9:**
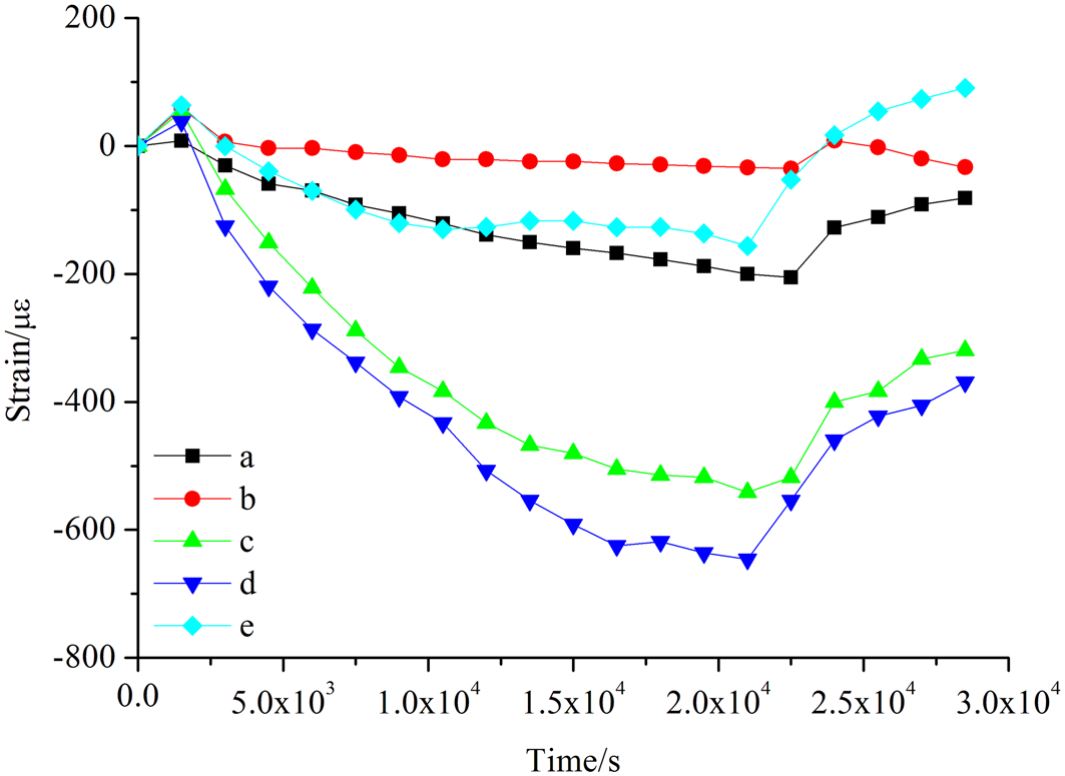
Strain–time relationship for steel tube inner wall.

The dynamic strain patterns at six measuring points a–f on the inner wall face of the hollow steel tube are shown in Fig. 9 (Measurement data from point f are abnormal and removed). At the beginning of the accelerated corrosion test, the a–f test points experience a short period of tension. The tension state in this early stage (Fig. 9) is mainly the effect of the thermal expansion of steel tube due to electric currents. When the test starts, the power is turned on to supply electricity for the accelerated corrosion process. At this moment, the steel tube has no rust but is heated to expand when electrons pass through. Consequently, overall, the steel tube is in tension state with the tensile forces generated. As the accelerated corrosion test proceeds, the energizing effect turns stable and more corrosives are produced due to chloride, which leads to the compression state. Therefore, these points turn into compression state. Overall, the compressive strain at each test point shows an increasing trend until the concrete completely cracks. At the beginning of the test, the growth rate of strain on each measuring point is faster. As the test proceeds, more rusty products are generated preventing the contact between corrosives and steel pipe, so the corresponding strain growth rate gradually decreases. Constraints from concrete to the steel tube vary along its longitudinal direction, and the two ends of the steel tube are subject to lower constraints while the middle experiences higher constraints. Consequently, the pressure strains at measurement points c and d which are at the middle of the inner wall of the steel tube are larger with higher growth rates. The concrete completely cracks at t = 2.2 × 10^4^ s. After cracking, the confinements from concrete on steel tube decreases, and the compressive strain of the steel tube declines rapidly. Declining rate is significantly higher than that of rising during the early stage of testing.

### Concrete surface strain field

**Figure 10:**
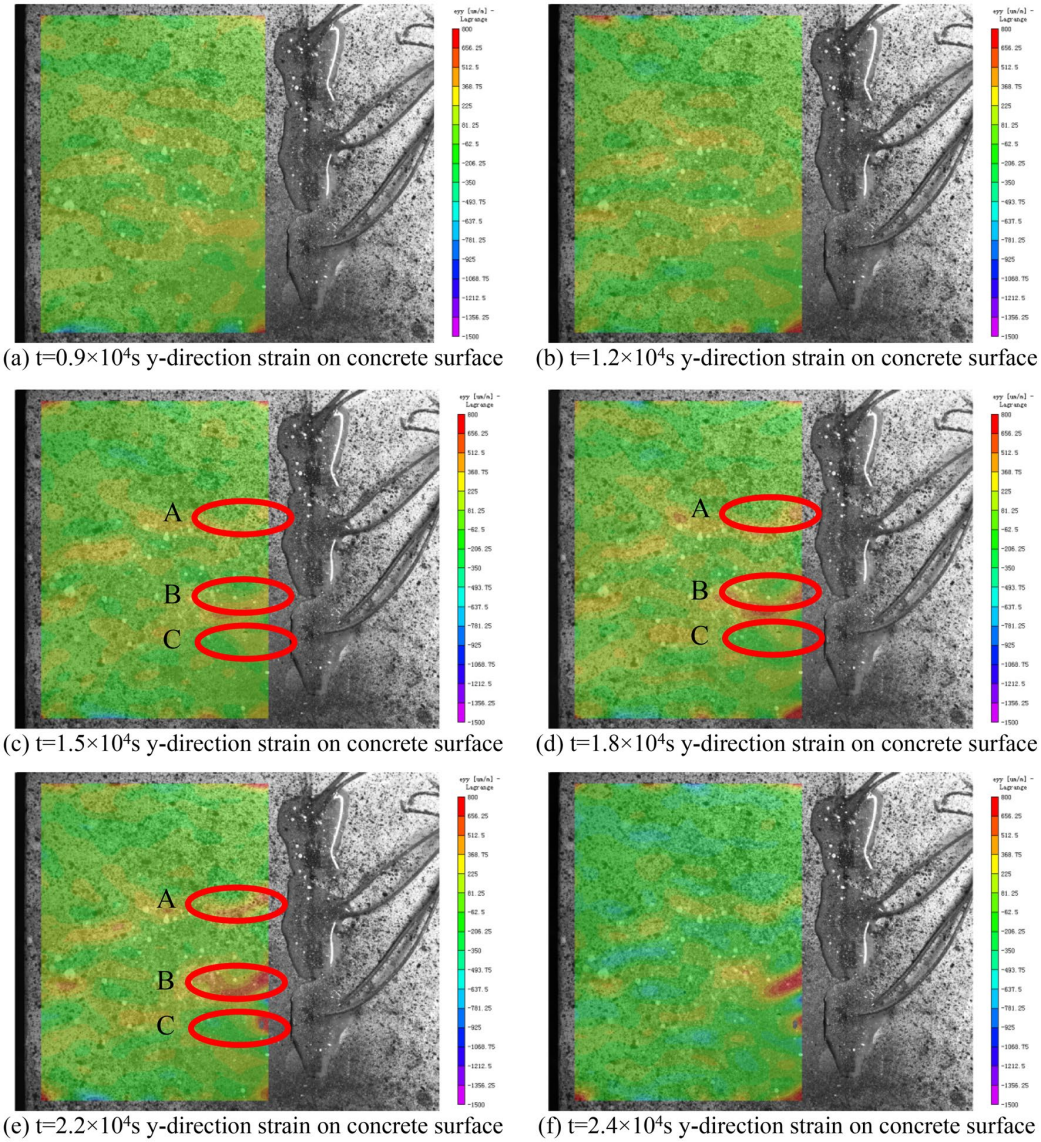
The y-direction strain on concrete surface.

The used strain gauge system can only measure the strains of a limited number of points on the concrete surface. To perform the full field analysis of the concrete surface strain, during the test, Basler CCD camera is used to collect the real-time strain information on concrete surface. The obtained figure series are then analyzed by the digital image correlation (DIC) method^17–19^. Figure 10 shows the y-direction strain field of the concrete specimen surface.

It can be seen that, at the beginning of the test (Fig. 10a,b), the strains along the y direction (perpendicular to the steel tube) on the concrete surface are relatively small and the impacts of rust expansion force are relatively low. It can be seen from Fig. 10c,d, as the time reaches t = 1.5 × 10^4^ s, tensile strains appear at three points A,B,C, and keep rising. When t = 2.2 × 10^4^ s in Fig. 10e, the strains at the three points arrive at their maximums and then the cracks present. Afterwards, the strains on the concrete surface redistribute, which can be seen from the strain field in Fig. 10f at t = 2.4 × 10^4^ s.

### Rebar corrosion rate

Theoretically, through Faraday's Law, the corrosion amounts of rebars can be described based on the magnitude of passing current. The mass of substance that experiences chemical changes on anode rebars is proportional to the amount of electricity passing through the electrode. The corrosion mass ∆m can be calculated as:

8 $$\Delta m = n \cdot M = \frac{Q \cdot M}{{F \cdot |z|}} = \frac{{M \cdot \int {I(t)dt} }}{F \cdot |z|}$$

where *n* is the amount of bar corrosion (in mol); *M* is the molar mass of iron (55.8 g/mol); *Q* is the quantity of electricity passing by anode (in C); I(t) is the external current intensity (in A) at time t (in s); *F* is Faraday constant (96,485 C/mol); *z* is the absolute valency of ion and the value for iron is 2.

The bar corrosion rate *ρ* is:

9 $$\rho = \frac{\Delta m}{m}$$

where m is the initial bar mass.

**Figure 11:**
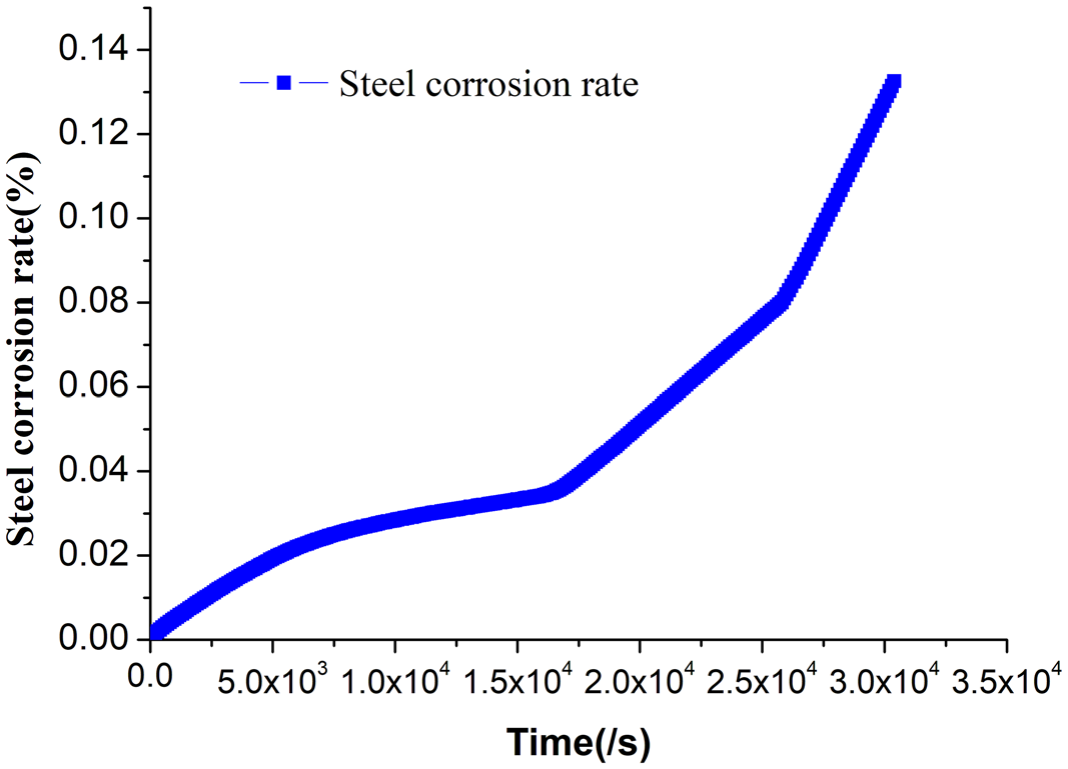
Change of corrosion rate of reinforcement with time.

By the above equation, the real-time bar corrosion rate can be obtained. As in Fig. 11, the corrosion rate of bar increases with the progress of concrete cracking. Bar corrosion dramatically speeds up after the concrete completely cracks. It indicates that concrete cracking has great impacts on bar corrosion.

### Verification of the relationship between the surface strain of concrete and the expansion force of reinforcement rust

**Figure 12:**
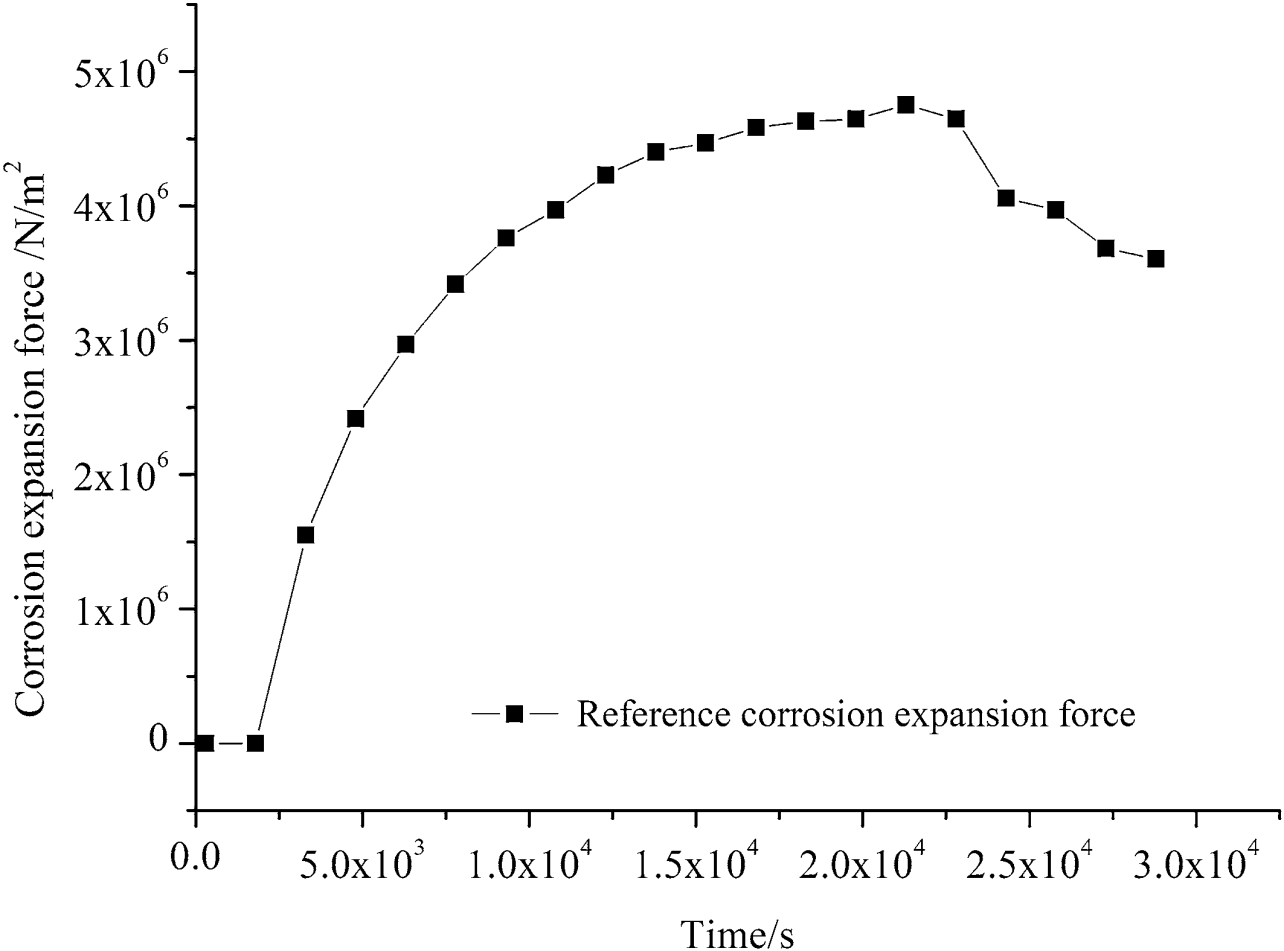
Referential corrosion expansion force versus time.

Strains on the inner wall of the steel pipe vary at different points, and so do the rust expansion forces associated with the outer wall. Therefore, the strain at measuring point C of the inner wall of the steel pipe in Fig. 5 is selected to derive the referential rust expansion force. Both point C and the corresponding strain gauge are 40 mm away from the end of the concrete and lie on the same cross section. The strain at point C is put into Formula (6), where *R* = 8 mm, r_1_ = 5 mm, E_S_ = 2.1 × 10^5^ MPa, μ = 0.3. The change pattern of rust expansion force versus time is shown in Fig. 12.

**Figure 13:**
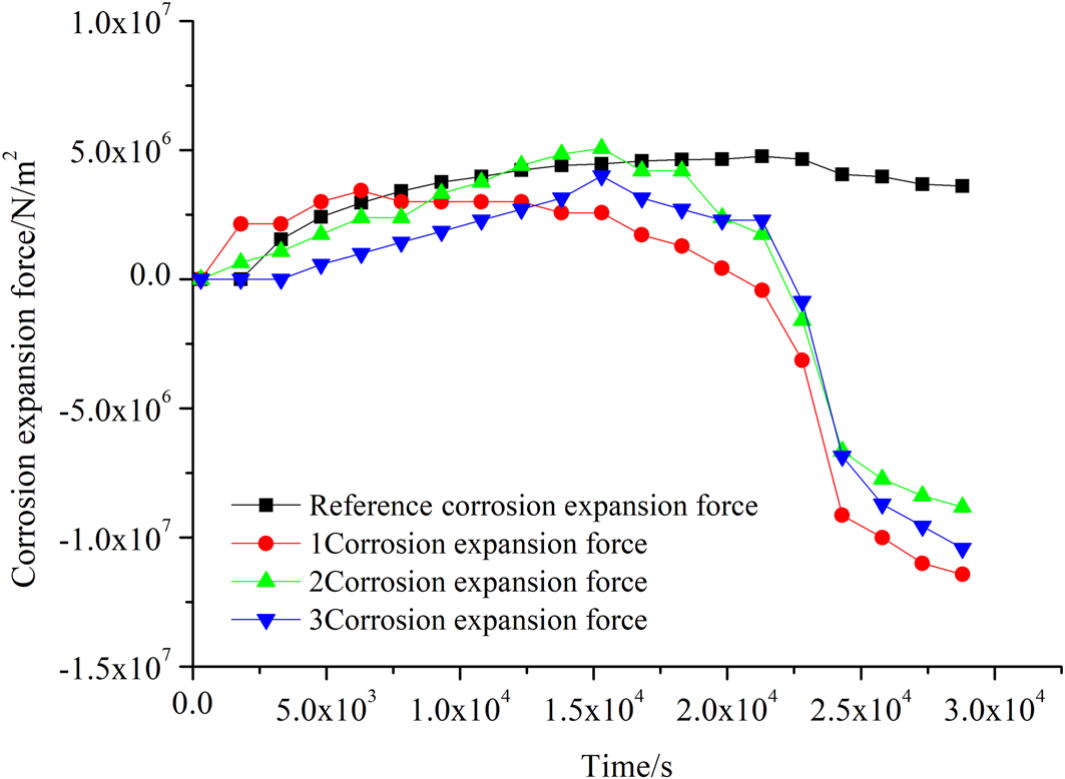
Comparison between referential corrosion expansion force and corrosion expansion force at different locations.

Putting concrete surface strain data into Formula (5) for calculation, where E_C_ = 3 × 10^4^ MPa, k = 0.6, μ = 0.3, a = 500 mm, the obtained results are plotted in Fig. 13. Before the concrete cracks appear, the rust expansion force values deduced from the strains at points 1, 2 and 3 are well consistent with the referential rust expansion force. After the appearance of the concrete cracks, the relationship cannot be simplified by Lame’s solution. Therefore, the rust expansion force derived from the concrete surface strain based on the established model gradually deviates from the referential rust expansion force until concrete is completely exposed. Consequently, it can be seen that the established model has a decent applicability in predicting the corrosion expansion force before the cracks appear in reinforced concrete structures.

## Conclusion and future work

In this paper, the prediction model for the rust expansion of the cuboid reinforced concrete specimen with square cross sections is established, and the accelerated corrosion test is carried out to study the relationship between the rust expansion force of reinforcement and the surface strain of concrete. Conclusions are as follows:

1. From the accelerated corrosion test, it can be found that the tensile strain on the concrete surface and the compressive strain on the inner wall of the reinforcement continue to increase prior to the appearance of cracks on the concrete surface. After the concrete cracks appear, the tensile strain of the concrete surface keeps decreasing. The concrete surface remains tensile until the concrete cracks completely. After complete cracking, the surface strain of concrete and the pressure strain of the inner wall of the reinforcement drop sharply, and the concrete surface changes from being tensile to be compressive.
2. A relationship model between the steel rust expansion force and the concrete surface strain is established based on the theory of elasticity. It is calibrated and verified by the physical experiment data. The model is found to be sufficiently valid for concrete before the cracks appear, but cannot be used to deduce the rust expansion force of reinforced concrete structure after the cracks appear.

The real-time prediction of the rust expansion force of reinforcement before concrete cracks remains important to the durability design of concrete structures. It can also provide critical information to forecast structure deterioration and estimate remaining service life for various life cycle engineering purposes^20^. The concise model proposed in this study can contribute to the quick prediction of the real-time rust expansion force in concrete structures to satisfy the above needs. In future, the research will be expanded to consider the non-uniform corrosion situation. Non-contact optical measurement methods will be introduced for calibrating theoretical models. Cohesive models and plastic models^21^ will also be considered for the rust expansion force prediction by comparing the advantages and disadvantages of different types of theoretical models.

It plans to study how the bar arrangements can affect the crack patterns, how the humidity can affect the cracking phenomenon, and how the cracks progress as time goes. Heating is simultaneously used to the current in this study. To use a compensation specimen which intends to eliminate the expansion effects due to heating is planned for conducting another series of experiments for comparison with our current study. The use of electrochemical techniques to measure corrosion will be considered.
